# Protocol for a Case-Control Study to Investigate the Association of Pellagra With Isoniazid Exposure During Tuberculosis Preventive Treatment Scale-Up in Malawi

**DOI:** 10.3389/fpubh.2020.551308

**Published:** 2020-11-26

**Authors:** Scott A. Nabity, Kelvin Mponda, Steve Gutreuter, Diya Surie, Anne Williams, Andrea J. Sharma, Elizabeth R. Schnaubelt, Rebekah E. Marshall, Hannah L. Kirking, Suzgo B. Zimba, Joram L. Sunguti, Laphiod Chisuwo, Mabvuto J. Chiwaula, Jesse F. Gregory, Robin da Silva, Michael Odo, Andreas Jahn, Thokozani Kalua, Rose Nyirenda, Belaineh Girma, James Mpunga, Nicole Buono, Alice Maida, Evelyn J. Kim, Laurence J. Gunde, Tigest F. Mekonnen, Andrew F. Auld, Adamson S. Muula, John E. Oeltmann

**Affiliations:** ^1^Division of Global HIV and Tuberculosis, U.S. Centers for Disease Control and Prevention, Atlanta, GA, United States; ^2^School of Public Health and Family Medicine, College of Medicine, University of Malawi, Blantyre, Malawi; ^3^National Center for Chronic Disease Prevention and Health Promotion, U.S. Centers for Disease Control and Prevention, Atlanta, GA, United States; ^4^McKing Consulting Corporation, Atlanta, GA, United States; ^5^Elizabeth Glaser Pediatric AIDS Foundation, Lilongwe, Malawi; ^6^National Public Health Reference Laboratory, Public Health Institute of Malawi, Lilongwe, Malawi; ^7^Department of Food Science and Human Nutrition, University of Florida, Gainesville, FL, United States; ^8^Department of HIV and AIDS, Malawi Ministry of Health, Lilongwe, Malawi; ^9^National Tuberculosis Control Program, Malawi Ministry of Health, Lilongwe, Malawi; ^10^Division of Global HIV and Tuberculosis, U.S. Centers for Disease Control and Prevention, Lilongwe, Malawi

**Keywords:** isoniazid, tuberculosis preventive treatment, tuberulosis, human immuno defficiency virus (HIV), pellagra, niacin (nicotinic acid), Malawi

## Abstract

**Background:** Pellagra is caused by niacin (vitamin B3) deficiency and manifested by a distinctive dermatitis. Isoniazid is critical for treating tuberculosis globally and is a component of most regimens to prevent tuberculosis. Isoniazid may contribute to pellagra by disrupting intracellular niacin synthesis. In 2017, Malawian clinicians recognized a high incidence of pellagra-like rashes after scale-up of isoniazid preventive treatment (IPT) to people living with HIV (PLHIV). This increase in pellagra incidence among PLHIV coincided with a seasonal period of sustained food insecurity in the region, which obscured epidemiological interpretations. Although isoniazid has been implicated as a secondary cause of pellagra for decades, no hypothesis-driven epidemiological study has assessed this relationship in a population exposed to isoniazid. We developed this case-control protocol to assess the association between large-scale isoniazid distribution and pellagra in Malawi.

**Methods:** We measure the relative odds of having pellagra among isoniazid-exposed people compared to those without exposure while controlling for other pellagra risk factors. Secondary aims include measuring time from isoniazid initiation to onset of dermatitis, comparing niacin metabolites 1-methylnicotinamide (1-MN), and l-methyl-2-pyridone-5-carboxamide (2-PYR) in urine as a proxy for total body niacin status among subpopulations, and describing clinical outcomes after 30-days multi-B vitamin (containing 300 mg nicotinamide daily) therapy and isoniazid cessation (if exposed). We aim to enroll 197 participants with pellagra and 788 age- and sex-matched controls (1:4 ratio) presenting at three dermatology clinics. Four randomly selected community clinics within 3–25 km of designated dermatology clinics will refer persons with pellagra-like symptoms to one of the study enrollment sites for diagnosis. Trained study dermatologists will conduct a detailed exposure questionnaire and perform anthropometric measurements. A subset of enrollees will provide a casual urine specimen for niacin metabolites quantification and/or point-of-care isoniazid detection to confirm whether participants recently ingested isoniazid. We will use conditional logistic regression, matching age and sex, to estimate odds ratios for the primary study aim.

**Discussion:** The results of this study will inform the programmatic scale-up of isoniazid-containing regimens to prevent tuberculosis.

## Introduction

Pellagra is a disease of niacin (vitamin B3) deficiency. Dietary sources of niacin include foods with bioavailable forms of niacin and protein-rich foods from which the amino acid tryptophan is converted to bioavailable niacin. Pellagra presents with a characteristic dermatitis (1). Other common symptoms include nausea, vomiting, and diarrhea. Advanced deficiency may produce painful oral lesions and neuropsychiatric effects, notably confusion and lack of motor control (e.g., weakness or ataxia). Untreated pellagra may result in death. There is no confirmatory laboratory test for pellagra.

Contemporary pellagra outbreaks have been reported in sub-Saharan Africa (2, 3), and the disease is endemic in Malawi (4). Undiversified consumption of unfortified, primarily subsistence maize, puts consumers at risk for niacin deficiency (5). Malawians are among the world's highest per capita maize consumers (6). In Malawi, ~15% of maize is centrally processed, and only about half of that stock is fortified (7); self-milled maize is rarely fortified. Foods rich in protein or bioavailable niacin (e.g., meat and nuts) are more expensive than locally milled maize and inaccessible to resource-limited households. Systematic pellagra surveillance does not occur in Malawi, but incidence appears to follow a seasonal pattern. During the period between sowing and harvesting the new maize crop, colloquially known as the lean season, many people in Southern and East Africa experience grain scarcity and restricted diets (8). In February 2017, Matapandeu et al. described nearly 700 persons with pellagra from one clinical catchment in the central region of Malawi during 2015–2016 (9). The study included few clinical or epidemiological details. However, the pellagra occurred in the setting of drought and high crop prices (10), and food insecurity was the presumed cause.

In the Malawi health system, referrals for pellagra-like skin conditions typically flow from peripheral health centers to district-level dermatology clinics and to any of four central hospitals (highest level of referral). Persons may also self-present to clinics at all levels. These clinics are staffed by mid-level clinicians with advanced training in dermatology and sexually transmitted infections (i.e., community dermatovenereologists) or physicians with advanced training in dermatology (11, 12). Hereafter, we collectively refer to these specialized clinicians as dermatologists.

Isoniazid preventive treatment (IPT) reduces tuberculosis incidence and deaths among people living with HIV (PLHIV) (13–17). In August–October 2017, Malawi scaled up daily IPT (adult dose: 300 mg of isoniazid plus 25 mg of pyridoxine [vitamin B6]) in five districts (18) with the goal of initiating approximately 300,000 PLHIV within 12 months. Within three months of rapid IPT scale-up, a dermatologist (co-author of this manuscript, KM) first reported an increase in pellagra incidence among PLHIV who recently initiated isoniazid. An informal clinician network passively reported additional pellagra from other facilities. This increase coincided with the onset of the lean season, which obscured epidemiological interpretations of this observation with respect to isoniazid.

Isoniazid has been implicated as a secondary cause of pellagra (19–23) by inhibiting the conversion of tryptophan to niacin (24). HIV infection disrupts tryptophan catabolism (25, 26) and may contribute to a niacin-deficient intracellular state (27, 28). Also, recent evidence from Malawi demonstrated lower levels of niacin in breastmilk of women with HIV who received protease inhibitor-containing antiretroviral therapy (ART) (29). Although current ART formulations in Malawi rarely include protease inhibitors (18), the potential impact and clinical significance of other regimens on niacin status are unknown. We hypothesized that isoniazid exposure may have contributed a final component of cumulative risks (i.e., baseline dietary deficiency and HIV infection and/or ART exposure) to convert subclinical niacin deficiency to clinically apparent niacin deficiency (i.e., pellagra). We present the protocol we developed to investigate the relationship between pellagra and isoniazid during the scale-up of IPT in Malawi. Access to our protocol details may assist other countries with similar investigations.

### Study Aims

Our primary aim is to determine whether exposure to isoniazid is associated with increased relative odds for pellagra while controlling for known pellagra risk factors. Secondary aims, which will provide supporting information but did not influence sample size requirements, include to:

- Measure the time from isoniazid initiation to first onset of pellagrous dermatitis.
- Compare the level of niacin metabolites (1-methylnicotinamide [1-MN] and l-methyl-2-pyridone-5-carboxamide [2-PYR]) in urine as a proxy for total body niacin status among subpopulations (i.e., according to risk exposure and pellagra status).
- Describe the 30-days clinical outcomes for persons with pellagra.

## Methods and Analysis

### Study Design

**Figure 1:**
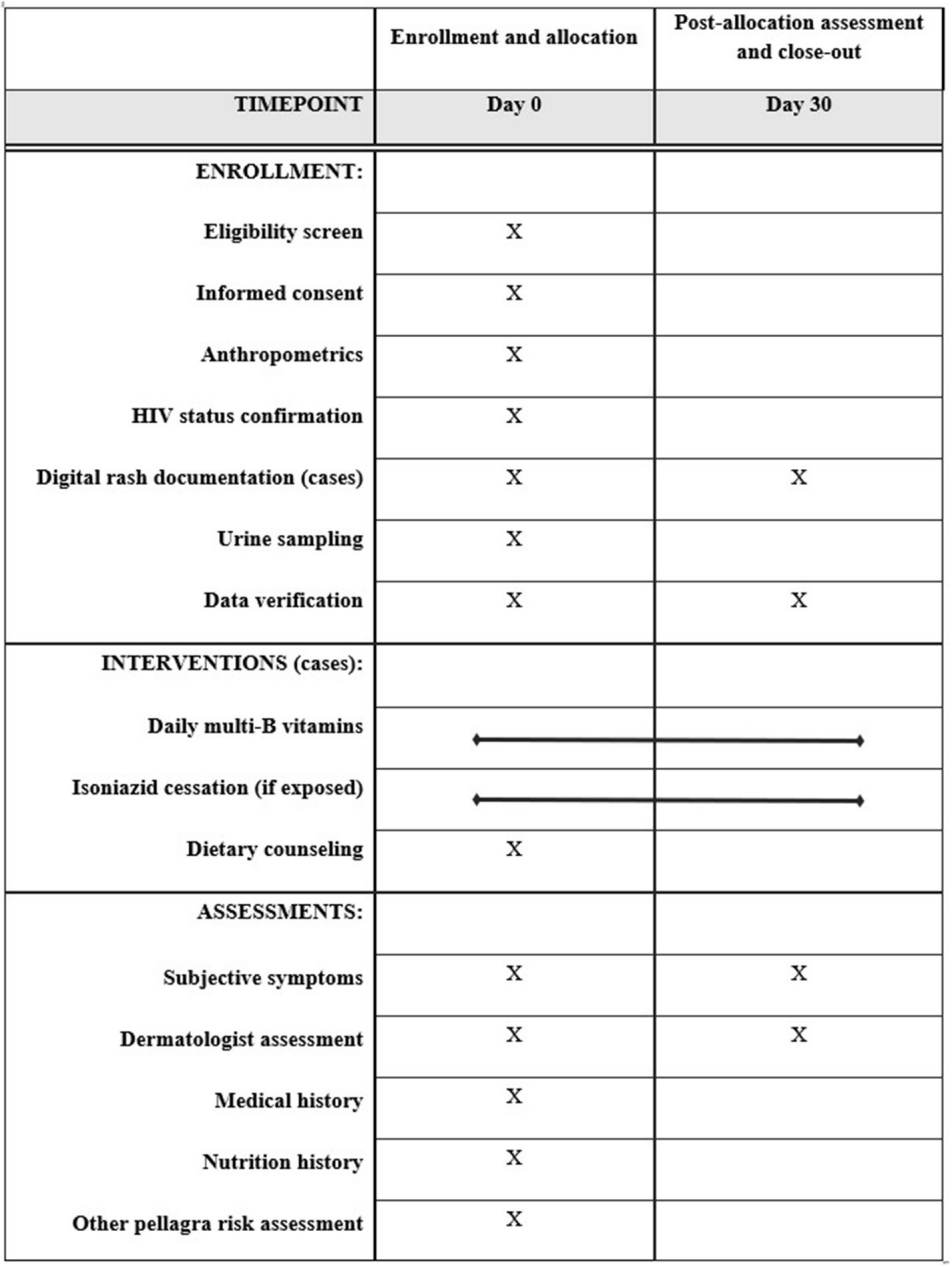
Standard Protocol Items: Recommendations for Interventional Trials (SPIRIT) enrollment, interventions, and assessments for the case-control protocol to study the association of pellagra and isoniazid in Malawi, 2019.

**Figure 2:**
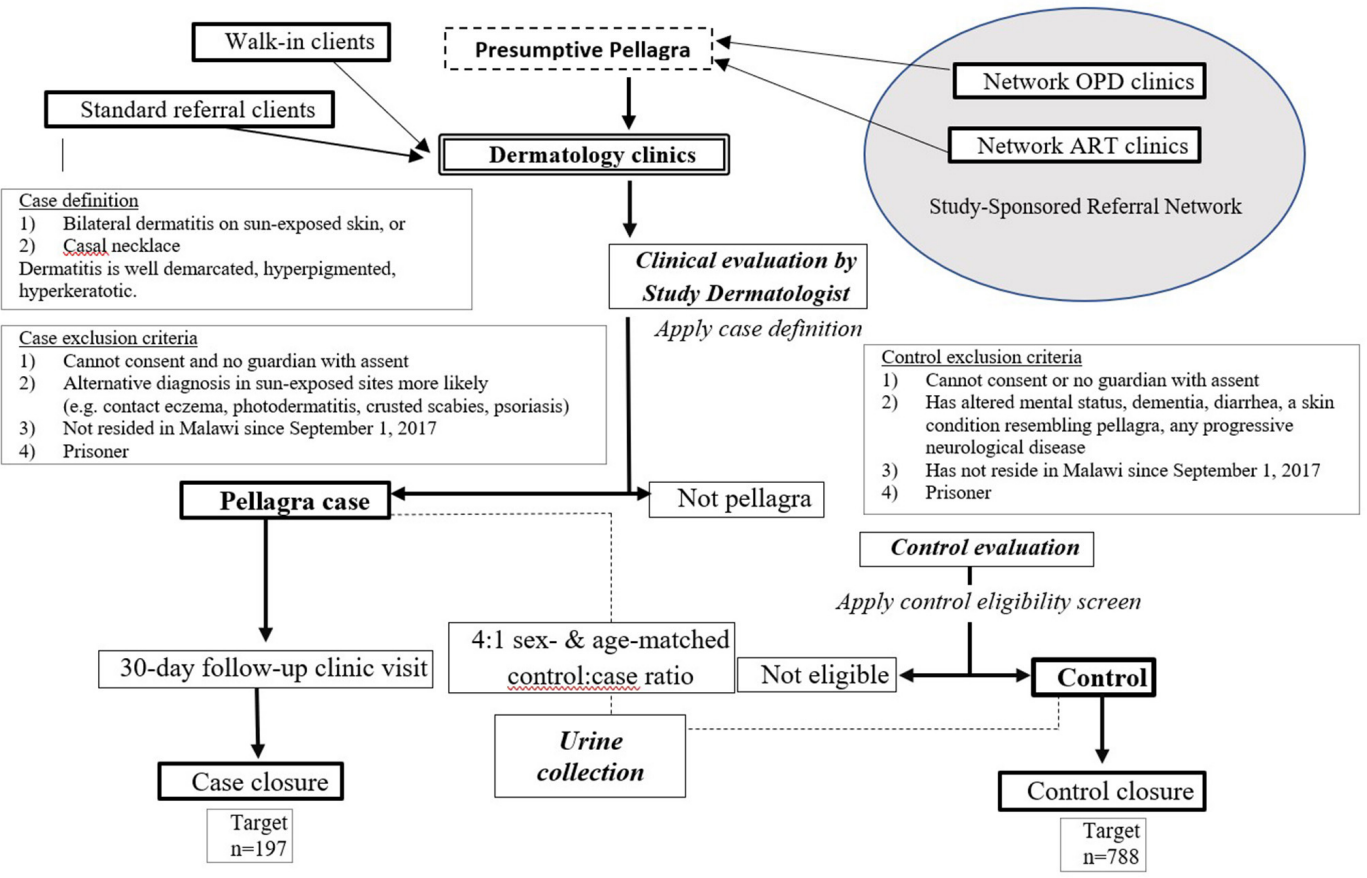
Flow chart of procedures for the case-control study of the association between pellagra and isoniazid exposure in Malawi, 2019.

This case-control study assigns four random controls per confirmed pellagra case-patient, matched by age and sex, within three dermatology clinics. The design was developed and is reported in accordance with standards for clinical trials per Standard Protocol Items: Recommendations for Interventional Trials (SPIRIT) (Figure 1 and Supplementary Table 1) (30). Figure 2 further details the study design. Details related to study sites, data collection tools, data management procedures, and informed consent materials may be requested from the corresponding author.

### Study Population and Setting

The target population consists of persons at risk for pellagra in three of the five IPT scale-up districts in Malawi (Lilongwe, Blantyre, and Zomba), selected for the presence of referral dermatology centers with dermatologists trained to diagnose pellagra. Enrollees are recruited from these purposively selected dermatology centers. We use September 1, 2017, when IPT was scaled up among PLHIV in the study area, as the initial date for possible isoniazid exposure. Study enrollment commenced in February 2019 and will proceed until the target sample is achieved, pending the results of a midpoint statistical analysis.

### Presumptive Pellagra Definition and Exclusion Criteria

Presumptive pellagra is defined as a rash on sun-exposed areas (e.g., face, feet, hands, forearms, or legs) or a distinctive plaque on the neck and/or chest (i.e., Casal necklace) of a person of any age. Characteristic pellagrous dermatitis is symmetrical, hyperpigmented, well-demarcated, and hyperkeratotic. A confirmed diagnosis of pellagra meets these criteria, and a specially trained study dermatologist rules out alternative diagnoses.

Exclusion criteria for participating in the study were:

1. Inability to provide consent and not having a parent/guardian to give permission (along with a concomitant child assent from those aged 7–17 years)
2. Not continuously residing in Malawi since September 1, 2017
3. Presence of an alternative diagnosis that might explain the rash in the exposed sites, morphologically or otherwise, as determined by study dermatologists (e.g., crusted scabies, sunburn, or contact eczema)
4. Prisoner.

### Case-Patient Recruitment

#### Presumptive Pellagra Referrals

**Figure 3:**
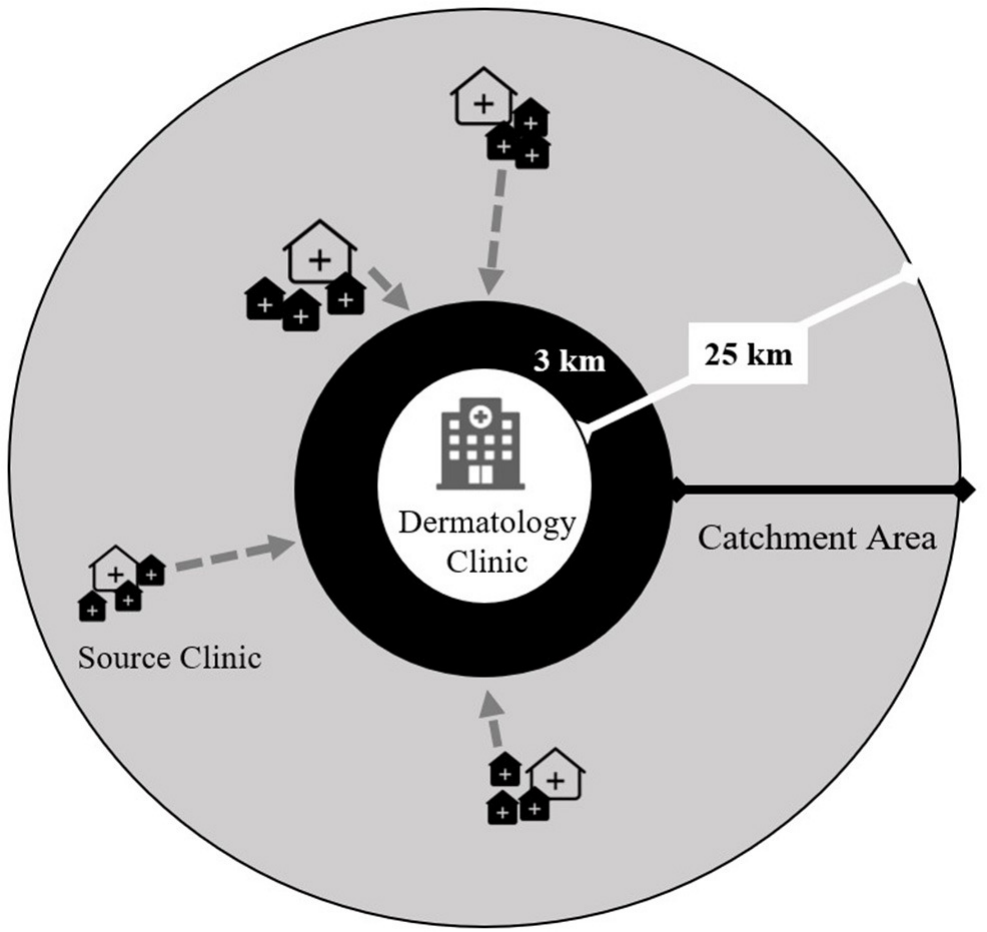
Study-supported source clinic referral network for persons with presumptive pellagra for the case-control study of the association between pellagra and isoniazid exposure in Malawi, 2019. The gray circle represents the catchment area for persons with presumptive pellagra. Each dermatology clinic was purposively selected within three of five districts where isoniazid preventive treatment was scaled up. The source clinics consist of randomly selected peripheral health centers that have both an HIV care clinic and general outpatient clinic and are located 3–25 km from the dermatology clinic. The locations and sizes of source clinic icons represent the varying distances from the dermatology clinic and the source clinics' patient volumes. The gray arrows show the flow of referrals of persons with presumptive pellagra from source clinics to the dermatology clinic, where experienced and study-trained dermatologists clinically assess referrals for study eligibility.

We trained staff at the main dermatology clinics in each of the selected IPT scale-up districts. We defined a 3–25-km radius catchment area around each clinic (Figure 3) and randomly selected three high-volume source clinics (peripheral health centers). Each source clinic has both an ART clinic and an outpatient department (OPD) on the same campus but does not have a dermatologist on staff. To enhance recruitment of participants representing the general population and limit selection bias based on HIV and isoniazid exposures, we trained staff from all departments within each source clinic to recognize pellagra-like rashes using a standardized job aid. The study reimburses the cost of patient transport and incentivizes clinicians to refer patients to the study. The study-supported structure of source clinics augments existing routine systems to refer skin conditions in Malawi but does not replace it. All persons diagnosed with pellagra at the dermatology clinics, regardless of referral pattern or walk-in status, are evaluated for study eligibility.

#### Dermatology Clinic Assessment

Persons without pellagra will receive standard of care for their skin condition per the Malawi Ministry of Health (MoH). For those with confirmed pellagra, we record a series of standardized digital, high-resolution photos, which are securely managed to ensure enrollee confidentiality. Case-patients also receive:

- Counseling on dietary considerations pertinent to pellagra.
- No-cost treatment for pellagra per World Health Organization (WHO) guidelines (1) with a multi-B vitamins containing 300 mg of nicotinamide daily (Table 1) provided by a trained study nurse. For enrollees weighing <20 kg, daily doses are one-third the standard formulation.
- Same-day confirmation of current HIV status per Malawi MoH guidelines (31) and same-day ART care to those with new HIV diagnoses.
- Instruction to discontinue isoniazid, if prescribed, per Malawi MoH recommendations.
- Instruction for PLHIV who are enrolled in ART care to visit their ART clinic within one week, including a document explaining the pellagra diagnosis and interventions.
- Instruction to return to the dermatology clinic in 30 days. At the follow-up appointment, participants receive a clinical assessment for pellagra cure, and the digital photographs are again recorded to document progress.

**Table 1:** Daily multi-B vitamin doses in the case-control study of the pellagra and isoniazid in Malawi, 2019.

**Micronutrient**	**Daily dose** **(Weight ≥ 20 kg^a^)**	**Daily dose** **(Weight < 20 kg)**
Nicotinamide (vitamin B3)	300 mg	100 mg
Thiamine (vitamin B1)	30 mg	10 mg
Riboflavin (vitamin B2)	30 mg	10 mg
Pyridoxine (vitamin B6)	9 mg	3 mg
Cobalamin (vitamin B12)	45 μg	15 μg
Calcium panthenate	150 mg	50 mg
Folic acid (vitamin B9)	4.5 mg	1.5 mg
Biotin (vitamin B7)	300 μg	100 μg
Ascorbic acid (vitamin C)	450 mg	150 mg

a *The total daily dose for persons weighing ≥20 kg is accomplished by administering one tablet three times daily (morning, noon, and bedtime). For persons weighing <20 kg, the daily dose is accomplished with one tablet administered once daily*.

### Recruitment of Control Participants

For each enrollee with pellagra, we sequentially match four control participants by age and sex from people with a skin problem other than pellagra who attend the same dermatology clinic. The groupings for age matching and stratification are 0–14 years, 15–24 years, 25–34 years, 35–44 years, 45–54 years, 55–64 years, and ≥65 years. We select control participants without considering the status of any risk factor, including HIV, ART, and isoniazid exposure status.

### Control Participant Exclusion Criteria

Control participants with one of the following criteria are excluded from the study:

- Inability to provide consent and not having a parent/guardian to give permission (along with a concomitant child assent from those aged 7–17 years)
- Skin conditions that may resemble pellagra when present in sun-exposed areas to limit misclassification (e.g., crusted scabies, photosensitive dermatoses, or psoriasis)
- Any of the following core signs or symptoms of pellagra:
  ◦ Diarrhea (≥2 loose stools daily)
  ◦ Altered mental status or dementia with onset since September 1, 2017
  ◦ Any progressive neurological disorder
- Not a continuous resident in Malawi since September 1, 2017
- Prisoner.

### Clinical and Epidemiological Data Collection

Isoniazid ingestion since September 1, 2017, the date that IPT was rolled out to participating districts, is the primary exposure of interest. Potential confounders include age, sex, HIV infection, ART use, an undiversified diet (i.e., consisting of primarily unfortified maize and little protein), and excessive alcohol consumption. Covariates and descriptors include:

- Medical history (including tuberculosis disease history)
- Pregnancy and lactation status (women)
- Parity (women)
- Nutritional history (see below)
- Food security status, per the Household Food Insecurity Access Scale (HFIAS) measurement tool (see below)
- Income status
- Anthropometric measurements (see below).

### Anthropometric Measurement

We perform anthropometric measurements for every pellagra case-patient and control participant. We directly measure weight (kg) using a calibrated, tare-capable digital balance with 200-kg capacity and precision to 0.1 kg (Smart Weigh; Chestnut Ridge, NY USA), and we measure height (cm) using a versatile stadiometer (PE-AIM-101, Perspective Enterprises; Portage, MI USA) fit for use on both adults and small children with accuracy of 3 mm. For children aged <2 years and non-ambulatory children, we measure height as supine head-to-heel length. From these measurements, we calculate BMI. We also measure mid upper arm circumference using an inelastic band (Seca; Chino, CA USA) for all enrollees.

### Case-Patient Follow-Up at 30 Days

We request all enrollees with pellagra to return to their enrollment site at 30 days to assess self-reported adherence to multi-B vitamin therapy and to obtain both subjective and objective measures of clinical improvement. For those taking isoniazid at the time of diagnosis, we also assess self-reported discontinuation. At enrollment, the study nurse sets a follow-up appointment date and provides an appointment reminder card. To further enhance retention at follow-up, a study nurse reminds patients of the appointment via phone call, we reimburse transportation costs to the clinic, and the study nurse attempts to contact enrollees who miss their scheduled appointments.

### Urine Sampling and Management

We request a subset of enrollees, as described below, to provide a urine sample for point-of-care isoniazid detection and/or niacin metabolite quantification. From consenting enrollees, we collect ≥50 mL of random urine and cool within 15 min. The study team transports specimens to the laboratory for processing within 4 h.

#### Point-of-Care Isoniazid Detection

Upon specimen receipt, the laboratory performs isoniazid detection assays (IsoScreen, GFC Diagnostics Ltd., Oxfordshire, UK) for all PLHIV (regardless of self-reported isoniazid status) and HIV-negative enrollees who report isoniazid exposure (i.e., multidrug tuberculosis disease treatment or child tuberculosis contacts) since September 1, 2017. Laboratorians do not have access to case-patient/control or exposure status and interpret the assay as positive, negative, or indeterminate at 5 min per manufacturer recommendations. For participants with current exposure to isoniazid, we anticipate the assay to have up to 95% sensitivity to detect isoniazid ingested within 30 h (32, 33).

#### Niacin Testing

The first 125 sequentially enrolled case-patients will provide urine samples for niacin metabolites, creatinine, and a point-of-care dipstick urinalysis for specific gravity (BTNX Rapid Response 10SG; Markham, ON Canada). One matched control participant per case-patient is also tested. Ultimately, we aim to compare niacin metabolite levels across the following enrollee strata according to pellagra status:

1. HIV-negative
2. PLHIV not receiving ART
3. PLHIV receiving ART (no isoniazid)
4. PLHIV receiving both ART and isoniazid.

Upon collecting urine samples from the initial 125 sequentially enrolled case-patients and 125 matching control participants, we will characterize the specimen donors with respect to the above strata. Because we enroll case-patients and control participants without considering their HIV, ART, or isoniazid exposures, we cannot predict the relative distribution of donor enrollees across strata. We expect, however, that some enrollee strata will be underrepresented (e.g., PLHIV not receiving ART and diagnosed with pellagra), so we may need to collect additional urine samples from ART, tuberculosis, or OPD clinics. We aim to have ≥20 enrollees in each analytic stratum. We will enroll these purposively sampled attendees strictly to compare the urine niacin metabolites across strata. Data from persons providing these extra samples will not be included in case-patient vs. control participant risk analyses.

### Niacin Metabolite Assay

Quantitative analysis of niacin metabolites 1-MN and 2-PYR will follow methods adapted from Creeke and Seal (34). The procedure uses high-performance liquid chromatography with ultraviolet detection. We will procure analytic standards commercially: 1-MN chloride (Sigma Chemical, M4627-1G; St. Louis, MO USA) and 2-PYR (Caymen Chemical, Nudifloramide; Ann Arbor, MI USA). Should 2-PYR be unavailable commercially, we will prepare it from 1-MN according to the method of Pullman and Colowick (35). To validate the assay, we will measure metabolite yields with and without known quantities of 1-MN and 2-PYR in 4 replicates and on at least 3 different days. We define acceptable performance by recovery as >95% for each 1-MN and 2-PYR, inter-day sample variance <5%, and inter-replicate variance <10%. We will run each analytic sample in duplicate. We document a detailed cold chain for samples stored at −20 to −80°C until analysis.

### Sample Size Calculation

Table 2 displays sample size requirements for plausible ranges of the minimal detectable odds ratio, power, and percentage of control participants exposed to isoniazid assuming 4:1 matching of control participants and case-patients. We will attempt to enroll at least 197 case-patients and 788 control participants to detect an odds ratio of at least 2.0 with 90% power, assuming isoniazid exposure as low as 12% among control participants (36). The range of values for isoniazid exposure assumes 80% IPT uptake among PLHIV in the target population (based on unpublished Malawi MoH program data) and a conservative estimate for HIV prevalence among dermatology clinic attendees of 15%−20%. We base the latter estimate on known HIV prevalence (~10% for rural and ~14% for urban Malawi) (37) and the expectation that the rate of HIV among skin clinic attendees is probably higher than the general population (38, 39). We do not need to account for loss to follow-up because the primary measures of association derive from data collected at enrollment. However, some observations of individual covariates may be unreported. We will use multiple imputation to address missing covariates for study enrollees to obtain completed datasets for analysis, with the assumption that data are missing at random or completely missing at random (40). We will assess key variables for interactions.

**Table 2:** Sample sizes and odds ratio for the case-control study of pellagra and isoniazid in Malawi, 2019.

**Odds ratio**	**Power (%)**	**Probability of isoniazid exposure among controls (%)**	**Needed cases**	**Needed controls**	**Total sample**
1.5	80	12	472	1,885	2,357
1.5	80	16	379	1,514	1,893
1.5	80	20	325	1,299	1,624
1.5	85	12	544	2,173	2,717
1.5	85	16	436	1,743	2,179
1.5	85	20	374	1,494	1,868
1.5	90	12	642	2,564	3,206
1.5	90	16	514	2,055	2,569
1.5	90	20	440	1,760	2,200
2.0	80	12	144	574	718
2.0	80	16	118	469	587
2.0	80	20	103	410	513
2.0	85	12	167	665	832
2.0	85	16	136	542	678
2.0	85	20	118	472	590
2.0	90	12	197	788	985
2.0	90	16	161	641	802
2.0	90	20	140	558	698

*The shaded values were those chosen for the sample size of this study*.

### Data Management and Analysis Plan

The data management team consists of a biostatistician, an epidemiologist, a medical epidemiologist, a database manager, a study coordinator, and two data entry clerks. We use REDCap Version 8.10.1 (Vanderbilt University; Nashville TN), with value range limits, for double data entry. The database manager performs regular data logic queries and feeds illogical results back to the study coordinator for joint remediation, where possible. Data are securely stored on redundant CDC servers. A supportive supervision team led by the study coordinator reviews paper documentation at each study site every 4–6 weeks for protocol adherence and data quality. We will use R Version 3.5.1, SAS Version 9.4 (SAS Institute Inc.; Cary, NC USA) or Stata 15 (StataCorp LLC; College Station, TX USA) for all analyses. Personally identifiable information, such as name and contact information, is maintained in a physically secured paper register by the Elizabeth Glaser Pediatric AIDS Foundation (EGPAF). Electronically stored information is managed using de-identified unique codes. Only named study investigators will have access to the final de-identified dataset.

#### Variable Definitions

We measure food security with a composite score from the validated HFIAS measurement tool (41). Because there is no validated dietary niacin consumption tool, we estimate recent dietary niacin intake using a 7-days food frequency questionnaire of 16 niacin-rich foods available in Malawi. Enrollees recall how many days they consumed each food item within the last week. We create a niacin score by summing food items across the numbers of days consumed times a food item multiplier. We base each food item multiplier on the presumed intake amount and its niacin content:

- If the quantity typically consumed is >100 g (e.g., rice, potato, or fortified maize) and the milligrams of niacin per 100 grams is ≥1.0, then the multiplier is 2.0.
- If the quantity typically consumed is ≤100 g (e.g., powdered coffee or groundnuts) and the milligrams of niacin per 100 g is ≥1.0, then the multiplier is 1.0.
- If the quantity typically consumed is >100 g (e.g., sweet potato, cassava, or unfortified maize) and the milligrams of niacin per 100 gram is <1.0, then the multiplier is 1.0.
- If the quantity typically consumed is ≤100 g (e.g., eggs or dairy), and the milligrams of niacin per 100 g <1.0, then the multiplier is 0.5.

We apply the WHO criteria of body mass index (BMI) <18.5 kg/m^2^ to classify adult men and non-pregnant adult women as underweight (42). For pregnant women, we will use mid upper arm circumference <23 cm to define underweight (43). For preschool (ages 6–59 months) and school-aged (ages 5–14 years) children, we will report stunting (length or height-for-age z-score <-2). For preschool children, we define acute malnutrition using a BMI-for-age z-score <-2.

Measuring a standard alcoholic drink in Malawi is challenging because consumption of home-brewed alcohol is extremely common (44), and the alcohol by volume of homebrew varies (45, 46). Consequently, we assess excessive alcohol consumption according to two components of the WHO Patterns of Drinking Score that we can readily capture: daily or nearly daily drinking and proportion of drinking events when the drinker feels drunk (47). We define excessive alcohol consumption as either drinking ≥10 days during the last 14 days, regardless of the number of drinks consumed per day, or feeling drunk ≥3 times in the last 14 days.

#### Primary Aim

**Figure 4:**
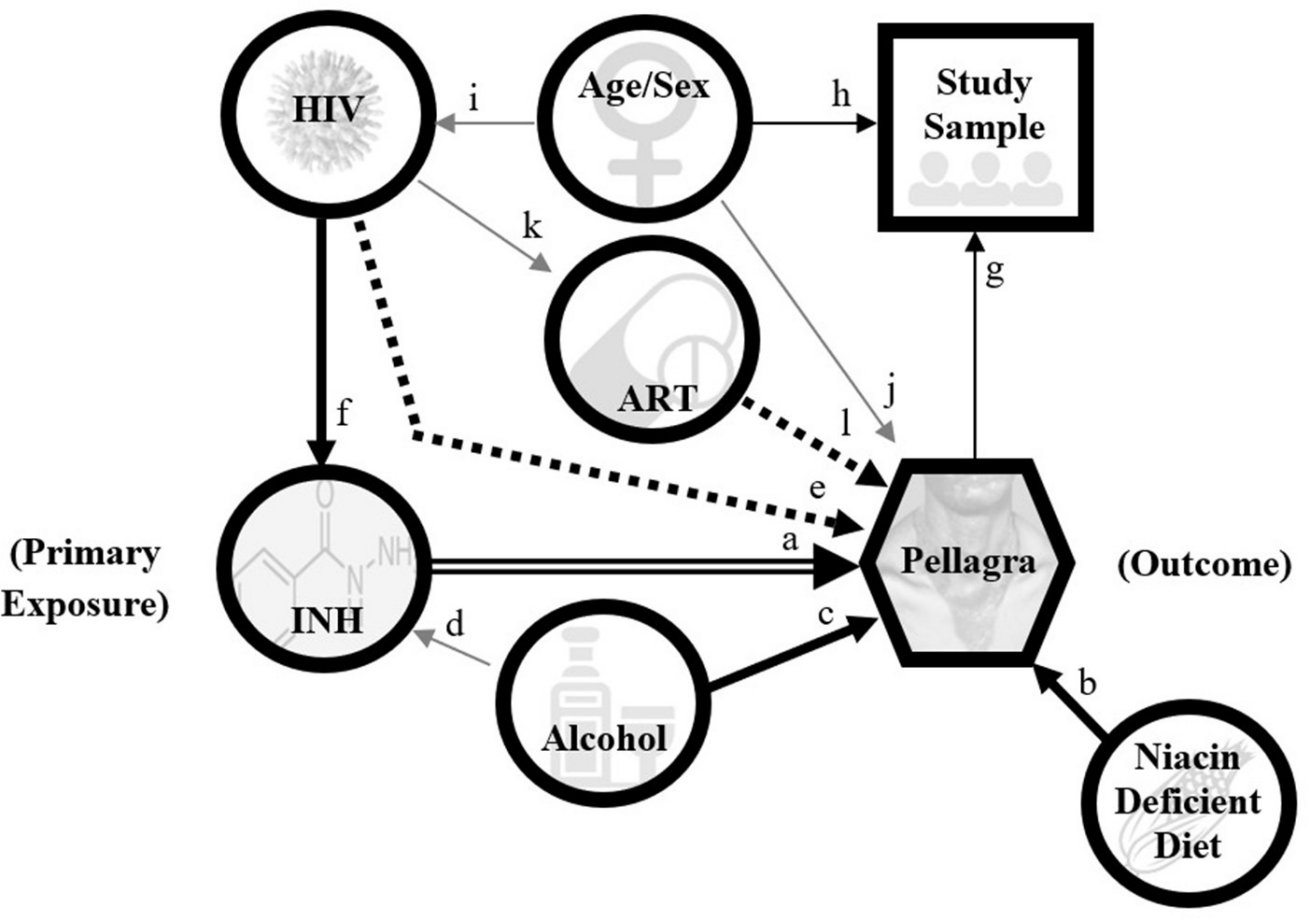
Directed acyclic graph model for pellagra to inform analyses in the case-control study of the association between pellagra and isoniazid exposure in Malawi, 2019. A causal relationship between isoniazid and pellagra is the main hypothesis (double arrow). An undiversified (unfortified maize-predominant), niacin-deficient diet is a known cause of pellagra (arrow b), as is excessive alcohol (arrow c). Excessive alcohol is also an exclusion to isoniazid preventive treatment (gray arrow d) per national guidelines due to risk of hepatoxicity and thus is a likely confounder. HIV may contribute to niacin deficiency via disruption of the tryptophan pathway; however, the association between HIV and pellagra remains uncertain (dotted arrow e). Because isoniazid preventive treatment is indicated for nearly all persons with HIV in the study area, HIV is positively associated with isoniazid exposure (arrow f). If HIV independently associates with pellagra, it will be a confounder. The study sample is selected on pellagra status (arrow g) and matched by age and sex (arrow h). HIV and pellagra are unevenly distributed by age and sex in Malawi (gray arrows i and j). Antiretroviral therapy (ART) is dependent on having HIV (gray arrow k), and speculatively may affect pellagra risk (dotted arrow l). This graphic does not depict all possible contributors to pellagra, including other medication effects.

We will approximate causal risk ratios for the effect of self-recalled isoniazid exposure on pellagra by odds ratios, according to the causal pathway model illustrated by Figure 4. The covariates related to HIV status (and thus ART use) and excessive alcohol are likely to be associated with both the outcome and exposure, as excessive alcohol can cause pellagra (48) and should preclude IPT initiation due to risk of hepatotoxicity (18). We will explore two analyses for estimating odds ratios. The first uses the product of conditional (on the age- and sex-matched strata) likelihoods in logistic regression (49). The full model will include the complete set of potential confounders. We will delete covariates for which *P* > 0.05 in sequential re-fittings to arrive at the most parsimonious model, which will then be the basis for inference about the effects on isoniazid exposure on pellagra. We will conduct a midpoint analysis of the primary aim using the Haybittle-Peto boundary (*P* < 0.001) to determine whether the study can be successfully concluded without full enrollment (50). A steering committee with government agency representatives, academics, programmatic implementation partners, and funders will monitor incident reports, potential adverse events, and data quality via weekly teleconference, and this committee will decide whether to continue the study based on results of the midpoint analysis. The steering committee will also coordinate the recommendation and submission of protocol amendments or other incidents as necessary.

#### Secondary Aims

We will calculate descriptive summaries for the duration (in months) from isoniazid initiation to onset of dermatitis. We also will calculate the spot urine 2-PYR/1-MN ratio (1), as well as the 1-MN/creatinine and 2-PYR/creatinine ratios in milligrams of urine niacin metabolite per gram of urine creatinine. Seal et. al. provided evidence that pellagra is associated with niacin deficiency and these metabolite-to-creatinine ratios capably detect niacin deficiency (51). We will assess the ratios for normality and analyze the quantified metabolites using box plots [minimum, maximum, mean with 95% confidence interval [CI], median, and interquartile range] according to nutrition status indicators, pellagra status, and hypothesized risk factors for pellagra. We will summarize clinical outcomes for persons with pellagra using basic descriptive statistics.

#### Stratifications

For primary analyses, enrollee recall defines isoniazid exposure. To ensure that an objective measure for isoniazid exposure concurs with self-report, we also will evaluate the model in the primary aim for the subset of patient-control participant pairings with isoniazid assay results. A visual interpretation of either positive or indeterminate will constitute a positive exposure to isoniazid. We also will stratify the primary analysis on referral type to the dermatology clinic (i.e., walk-in vs. referral by a clinician) and recency of isoniazid exposure (i.e., within two months before diagnosis vs. any time after September 2017).

### Ethics

The University of Malawi College of Medicine Research and Ethics Committee (reference P.10/18/2512), the Advarra Institutional Review Board (reference EG0223), and the CDC Human Research Protection Office (reference 7173) approved the study. All enrollees (and/or their guardians) undergo an informed, signed consent/permission process by a trained study nurse. Children aged 7–17 years must assent with a signature or thumb print. Participants can agree to all or some of the terms of the study. We enroll adults with altered mental status (AMS) if a first-degree relative is agreeable. The process for people with AMS is similar to that for minors aged <18 years, and we solicit assent from those with AMS when possible. Illiterate persons may provide a thumbprint alongside a witness signature after having the consent form read. We translated study questionnaires and patient educational materials to Chichewa to ensure cultural accuracy.

Participation involves minimal risk. Benefits of participation include knowing current HIV status and receiving WHO-recommended treatment for pellagra. The global community may benefit from the knowledge gained regarding the risk and treatment of pellagra among people prescribed isoniazid. Additionally, we compensate study participants for their time as potential income lost based on the local minimum wage (2,000 Malawian Kwacha [~US$2.75] per clinic visit). We reimburse transportation costs for persons with presumptive pellagra referred to a dermatology clinic and case-patients with confirmed pellagra returning for 4-weeks follow-up. To ensure scientific integrity, participant safety, and protocol adherence, an audit will be performed by senior scientists from EGPAF, independent of the investigators.

## Discussion

This study investigates the relationship between isoniazid and pellagra. We chose a case-control design due to the anticipated rarity (<1%) of the outcome among those exposed to isoniazid and the multiple potential contributors to pellagra in the target population. A prospective approach would therefore require a large number of enrollees. Because the districts in Malawi in this study had nearly achieved saturation of IPT initiations among PLHIV already in care at study initiation, prospective enrollment would also depend largely on PLHIV newly enrolled in care, further prolonging the observation period. We will complete a case-control study much more rapidly to produce actionable results for public health intervention.

We further apply several strengthening features to our case-control design. First, we quantify urine niacin metabolites to associate niacin deficiency with the outcome of interest (i.e., pellagra) and key exposures. We also use a point-of-care urine assay to affirm the primary exposure, isoniazid ingestion. Because there is no laboratory confirmation for pellagra, the study uses a detailed definition, standardized rash documentation, and expert diagnosis by specially trained dermatologists. Further, to account for potential confounders, we use a detailed questionnaire that includes 7-days food frequency, household food insecurity, alcohol consumption, and clinical history. Lastly, the study follows persons with pellagra for 30 days to document response to multi-B vitamin treatment containing nicotinamide (alongside isoniazid discontinuation, if exposed).

The study design has limitations. All case-control studies are subject to selection bias, preclude estimation of community incidence, and may limit causal inference (52). Nonetheless, our causal hypothesis between isoniazid and pellagra is supported by biologically plausible evidence. We aim to reduce selection bias regarding HIV and isoniazid status by supporting a study-specific referral mechanism from randomly selected peripheral health centers while maintaining the regular clinic flow of patients at the dermatology recruitment sites. Because the study involves clients obtaining standard clinical care, we cannot blind dermatologists to patient HIV status or isoniazid exposure history. We expect the risk of isoniazid-associated pellagra to be low, and the study may be underpowered to detect a small but significant odds ratio (<2.0). To limit recall bias, we review the enrollee's medical record for confirmation and use prompts to elicit medications; nonetheless, we expect any bias to be non-differential between case-patients and controls. Lastly, baseline dietary patterns in the target population may not differ enough for meaningful analytic stratification of niacin intake using an exploratory dietary niacin consumption tool.

This is the first hypothesis-driven epidemiological assessment of the association between isoniazid and pellagra. Isoniazid is critical for treating and preventing tuberculosis and controlling this global epidemic. In September 2018, the United Nations General Assembly declared a goal to end tuberculosis (53), which includes the administration of tuberculosis preventive therapy to 30 million people (including six million PLHIV) by 2022, and these regimens will contain isoniazid. In conjunction with the steering committee, we will disseminate the findings to stakeholders for policy development and publish findings.

## Conclusion

The results of this study will inform the programmatic scale-up of isoniazid-containing regimens to prevent tuberculosis.

## Ethics Statement

This study involved human participants were reviewed and approved by Centers for Disease Control and Prevention University of Malawi College of Medicine Advarra. Written informed consent to participate in this study was provided by the participants' legal guardian/next of kin.

## Author Contributions

SN, KM, SG, DS, AW, AS, ES, HK, AJ, TK, LG, AA, ASM, and MO conception of study. SN, KM, SG, DS, AW, AS, ES, HK, AJ, LG, ASM, and MO design of study. SN, KM, SG, JG, and RS drafted protocol. SG, DS, AW, AS, ES, RM, HK, SZ, JS, LC, MC, JG, RS MO, AJ, TK, AM, EK, LG, TM, AA, and ASM significantly contributed to improvement of the protocol. SN, KM, SG, DS, AW, AS, ES, RM, HK, SZ, JS, LC, MC, JG, RS, MO, AJ, TK, RN, BG, KM, JM, NB, AM, EK, LG, TM, AA, ASM, and MO reviewed and approved final protocol. All authors contributed to the article and approved the submitted version.

## Conflict of Interest

AW was employed by the company McKing Consulting Corporation. The remaining authors declare that the research was conducted in the absence of any commercial or financial relationships that could be construed as a potential conflict of interest.

## Abbreviations

1-MN: 1-methylnicotinamide
2-PYR: l-methyl-2-pyridone-5-carboxamide
AMS: Altered mental status
ART: Antiretroviral therapy
BMI: Body mass index
CDC: United States Centers for Disease Control and Prevention
CI: Confidence interval
EGPAF: Elizabeth Glaser Pediatric AIDS Foundation
HFIAS: Household Food Insecurity Access Scale
HIV: Human Immunodeficiency Virus
IPT: Isoniazid preventive treatment
MoH: Ministry of Health
OPD: Outpatient department
PEPFAR: President's Emergency Plan for AIDS Relief
PLHIV: Persons living with HIV
SPIRIT: Standard Protocol Items: Recommendations for Interventional Trials
WHO: World Health Organization.
